# Disaccharides: Influence on Volatiles and Phenolics of Sour Cherry Juice

**DOI:** 10.3390/molecules22111939

**Published:** 2017-11-09

**Authors:** Emil Zlatić, Anita Pichler, Mirela Kopjar

**Affiliations:** 1 Biotechnical Faculty, Jamnikarjeva 101, 1000 Ljubljana, Slovenia; emil.zlatic@bf.uni-lj.si; 2 Faculty of Food Technology, Josip Juraj Strossmayer University in Osijek, F. Kuhača 20, 31000 Osijek, Croatia; anita.pichler@ptfos.hr

**Keywords:** sour cherry juice, sugar addition, flavor, phenolics

## Abstract

The food industry is continuously developing ingredients, processing methods and packaging materials to improve the quality of fruit products. The aim of this work was to study the effect of sugars, a common ingredient in the food industry, on phenolics and volatiles of sour cherry juice. Sucrose, trehalose and maltose chemical isomers were chosen for this investigation. All sugars influenced the evaluated parameters. Samples with maltose addition had lower, while samples with sucrose and trehalose addition had higher anthocyanin content than the control sample. Generally, trehalose had a higher positive effect on volatiles with the desired flavor note.

## 1. Introduction

Food products are a complex matrix consisting of volatile and non-volatile compounds. Chemical composition, the macro- and micro-structure of foods and particle size are known to influence the volatile and non-volatile amount [1]. The characteristic flavor and color of fruits and their products greatly contributes to their overall acceptance by the consumer. The fragility and high post-harvest respiration rate of fruits results in nutritional and microbiological deterioration and limited shelf-life, as well as a loss of quality and health benefits. To minimize these negative effects, fruits are converted into frozen, dried, and canned products, or processed into jams, jellies, and juices for longer storage to satisfy various markets and consumer demands [2,3]. It is important that fruit properties are transferred from the raw material to the product. Nevertheless, during processing, chemical and biochemical changes of characteristic compounds cannot be avoided, causing a change of the color and aroma of fruit products. The flavor of fruits and fruit products is the result of a mixture of different volatile compounds. The equilibrium between the different volatile compounds and their concentrations are responsible for the overall flavor profile of fruits and their products [4]. The flavor of sour cherries is composed of a great number of organic components, including carbonyls, alcohols, esters, acids and terpenes. Next to benzaldehyde, which is described as a character-impact compound, the important aroma compounds of sour cherry are benzyl alcohol, 2-phenyl ethyl alcohol, eugenol, 2-hexanal, α-ionon and β-ionon [5,6]. Besides the aroma profile, color is one of the main distinguishing characteristics between products. Anthocyanins are responsible for the red color of sour cherry. Sour cherry is rich in phenolic compounds with substantial quantities of anthocyanins, in addition to hydroxycinnamates, flavonols, and flavan-3-ols[procyanidins] [7,8,9,10,11]. These compounds, as other polyphenols, are natural antioxidants. Antioxidants have potential health benefits and antioxidant activity is one of the most considered features in the definition of food nutritional quality. There are more and more studies associating antioxidant-rich diets with a lower incidence of cardiovascular disease, cancers, and age-related degenerative processes [12]. Sucrose is a commonly-used sugar in fruit product formulation. Next to sucrose, maltose and trehalose, two sugars that have a lower sweetness than sucrose, were chosen for this study. Also, all selected sugars are chemical isomers. The influence of sugars on fruit product quality has been the subject of many studies [13,14,15,16,17,18,19,20,21,22,23,24]. The positive influence of trehalose, as a replacement or partial replacement of sucrose, on the retention of volatile compounds has been proven in different products and semi-products such as strawberry cream fillings, strawberry puree, pear products, and freeze-dried sour cherry puree [13,14,15,16,17,18,19]. Also, a positive influence has been proven on phenolics, anthocyanins and antioxidant activity in mandarin juice, blackberry juice, freeze-dried sour cherry puree, freeze-dried apple puree, and strawberry cream fillings [19,20,21,22,23,24].

The aim of this study was to evaluate the influence of the addition of disaccharides (sucrose, maltose and trehalose) to sour cherry juice. Phenolic compounds, anthocyanins and antioxidant activity were investigated in order to evaluate the influence of the mentioned sugars on the color of sour cherry juice, but also on the possible health benefits of prepared products. For consumer acceptance, the flavor of products is very important, thus the volatile profile of prepared products was investigated so that disaccharide impact on both color and flavor can be estimated.

## 2. Results

### 2.1. Evaluation of Flavor

**Figure 1:**
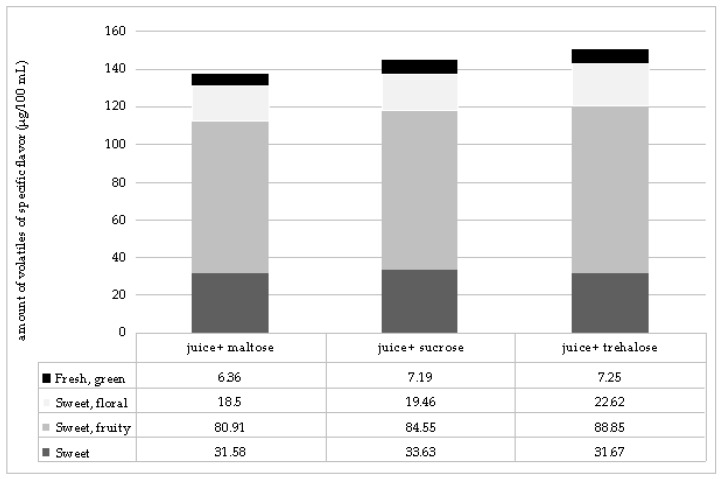
Amount of volatiles of specific flavor notes in sour cherry juice without and with addition of disaccharides.

Analyses of the volatiles of specific flavors revealed that sugar had a high impact on the overall flavor profile (Figure 1). Fresh, green volatiles (hexyl-acetate and 1-hexanol) were determined in the highest amount in the samples with sucrose and trehalose, 11% higher than in juice without sugar addition. Sweet, floral volatiles (α-ionone, β-ionone, benzyl alcohol, 2-decanon, geranic oxide, β-cyclocitral) were slightly lower in juice with sucrose and maltose addition, while in the samples with trehalose addition a higher amount of those volatiles was determined (8%). In all samples with sugar addition, sweet, fruity volatiles (benzaldehyde, 2-hexenal, 3-methyl-3-buten-1-ol, 2-methyl buthyl butanoate, 3-methyl buthyl butanoate, 2-heptanol, 1-octanol, 1-butanol-3-metyl-acetate) were determined in lower amounts than in juice without sugar addition. Comparing only sugars, samples with trehalose had the highest amount of those compounds. The lowest amount of pungent volatiles (acetic acid, 1-pentanol, 1-penten-3-ol) was determined in juice, and with addition of sugars, their amount increased. On the contrary, ethereal, alcoholic compounds (1-propanol, 2-methyl-1-propanol, 3-methyl-1-butanol, 1-hexanol, 2-methylbutanoate, 2-ethyl hexyl acetate) were determined in the highest amount in juice without sugar addition, and with the addition of sugars their amount decreased. In the case of sweet volatiles (diacetyl, 1-butanol, acetoin, eugenol), there was no difference between juice with the addition of trehalose or maltose and juice, while with the addition of sucrose slightly higher values were obtained.

The results of individual volatile amounts in sour cherry juice with the addition of sugars are presented in Table 1. Considering the ester group, five esters were identified, namely 1-butanol-3-methyl-acetate, hexyl acetate, 2-methyl butanoate, 2-methylbuthylbutanoate and 3-methylbuthylbutanoate. In all cases, the addition of sugars had an effect on the ester amount. In the case of both acetate esters, the addition of maltose didn’t cause an increase in their amount in comparison to the control sample. Samples with the addition of sucrose or trehalose had higher amounts of both acetate esters, with trehalose having a higher positive effect. Both esters have a sweet, fruity, fresh note. The investigated butanoate esters have a fruity note with a creamy, buttery undernote. The addition of sucrose had no effect on 2-methyl butyl butanoate (1 µg/100 mL), while in samples with maltose or trehalose addition, a higher amount was detected (1.3 µg/100 mL). Similar results were obtained for 3-methylbuthylbutanoate, samples with sucrose and trehalose addition had a higher amount of these esters (0.231 and 0.290 µg/100 mL, respectively) than the control sample and the sample with maltose addition (0.2 µg/100 mL). The addition of sugars had a positive influence on 2-methyl butanoate. The 2-methyl butanoate amount in samples with sugar addition was 1.9 µg/100 mL in comparison to the control sample, 1.616 µg/100 mL. 1-propanol and 2-methyl-1-propanol alcohols that are described by an ethereal, fusel-like note, in the control sample were determined in amounts of 4.365 and 14.335 µg/100 mL, respectively. The addition of sugars affected the amount of both alcohols. The sample with the addition of maltose had the lowest amount of 1-propanol (3.923 µg/100 mL) and the sample with sucrose addition the highest amount (4.472 µg/100 mL). All samples with sugar addition had a lower amount of 2-methyl-1-propanol in comparison to the control sample. Among samples with sugar addition, the sample with the addition of maltose had the lowest amount of this alcohol (9.079 µg/100 mL) and the sample with sucrose addition the highest amount (12.0258 µg/100 mL). Compering butanol derivates, which are described by a sweet, fruity but fusel, alcoholic note, it can be observed that the amount of 1-butanol is the same in all samples (29.5 µg/100 mL), except in the sample with sucrose addition, in which a slightly higher amount (31.331 µg/100 mL) of this alcohol was determined. In the case of the other two determined derivates, all samples with sugar addition had lower amounts of those alcohols in comparison to the control sample. The 1-pentanol (described by a pungent, fermented, fusel like note) was determined in all samples with sugar addition in higher amounts than in the control sample (1.163 µg/100 mL). Samples with maltose or sucrose addition had higher amounts of this alcohol, 1.9 µg/100 mL, than the sample with trehalose, 1.615 µg/100 mL. There was no difference in the amount of 1-penten-3-ol between samples with the addition of sugars in comparison to the control sample. The fruity and alcoholic compound, 1-hexanol, was determined in higher amounts in samples with addition of sucrose or trehalose (6.2 µg/100 mL) than in the control sample and the sample with the addition of maltose (5.7 µg/100 mL). The 2-heptanol, characterized by a fresh, fruity note, was determined in higher amounts in samples with sugar addition. The floral with a sweet, fatty nuance alcohol 1-octanol was determined in the highest amount in the sample with sucrose addition (1.032 µg/100 mL) and in the lowest amount in the sample with maltose addition (0.469 µg/100 mL). Benzyl alcohol and 2-phenyl ethyl alcohol are compounds that are responsible for the characteristic flavor of sour cherries. Benzyl alcohol, which is characterized by floral, rose, phenolic notes, was determined in the control sample in the amount of 3.477 µg/100 mL. The addition of sugars highly affects this volatile compound. In the sample with maltose addition, very low amounts of benzyl alcohol were determined, only 0.0048 µg/100 mL, while in samples with sucrose or trehalose addition, a higher amount (4.1 µg/100 mL) in comparison to the control was determined. Phenyl alcohol, which is also characterized by a floral rose note, was determined in much higher amounts in the control sample, 3.556 µg/100 mL, than in samples with sugar addition. Even if sugars didn’t have positive effect of 2-phenyl ethyl alcohol, the highest amount was determined in samples with sucrose and trehalose, 0.43 µg/100 mL. Benzaldehyde, one of the most characteristic sour cherry volatile compounds, has a strong, sharp, sweet, bitter, almond, cherry-like flavor note. All samples with the addition of sugars had a lower amount of benzaldehyde than the control sample (85.901 µg/100 mL). Comparing the influence of sugars, trehalose had the highest positive influence on these characteristic compounds. Next to benzaldehyde, a very important aldehyde in the characterization of sour cherry aroma, is 2-hexenal, that is described as having a sweet, almond, fruity note. While the addition of maltose had no effect on 2-hexenal (0.45 µg/100 mL), the addition of sucrose and trehalose resulted in higher amounts of this volatile compound (0.51 µg/100 mL). The same effect was observed for β-cyclocitral. In the case of α-ionone and β-ionone (compounds with a woody, sweet, fruity note), it was observed that only the sample with the addition of trehalose had similar content to the control sample, while samples with sucrose or maltose addition had lower amounts of those two volatile compounds, which are also important for the flavor of sour cherries. Diacetyl and acetoin, volatiles described by a sweet, creamy, buttery note, as well as 2-decanon (orange, floral, fatty note) were determined in the same amount in samples with sugars as in the control sample. Sugars had a high impact on geranic oxide, a volatile that is a sweet floral with a woody, cooling nuance. The amount of geranic oxide was around 8 µg/100 mL in samples with trehalose and maltose, 4.969 µg/100 mL in sample with sucrose addition, and only 2.991 µg/100 mL in the control sample. Eugenol (sweet, spicy, woody volatile) was determined in higher amounts in samples with sugar addition (0.6 µg/100 mL) in comparison to the control sample (0.473 µg/100 mL).

### 2.2. Evaluation of Phenolics

The results of the determination of the phenolics, flavonoids and flavonols of sour cherry juice with the addition of sugars are presented in Table 2. The phenolic content in the control sample was 1.550 g GAE/L. Samples with the addition of sucrose and trehalose had a higher phenol content, 1.600 and 1.633 g GAE/L, respectively, in comparison to the control sample. With the addition of maltose, the phenolic content decreased, 1.443 g GAE/L. Considering flavonoid and flavanone content, a different trend was observed. The flavonoid content in the control sample was 354.84 mg RE/L. Samples with the addition of sucrose and maltose had higher flavonoid contents than the control sample. The sample with the addition of maltose had the highest flavonoid content, 378.49 mg RE/L. In the case of trehalose addition, the lowest flavonoid content was observed, 330.19 g RE/L. The flavonol content in the control sample was 121.11 mg RE/L. Samples with the addition of sucrose and trehalose had lower flavonol content (79.84 mg RE/L and 28.33 mg RE/L, respectively), while the sample with the addition of maltose had a higher flavonol content (161.98 mg RE/L) than the control sample.

The anthocyanin content and the percentage of polymeric color of sour cherry juice with the addition of sugars are presented in Table 3. The anthocyanin content in the control sample was 214.038 mg C-3-G/L. The addition of all sugars had an effect on anthocyanin content. Samples with the addition of sucrose and trehalose had higher anthocyanin content and samples with the addition of maltose had lower (206.649 mg C-3-G/L) anthocyanin content than the control sample. Trehalose addition had a higher effect than sucrose. The sample with trehalose addition had 297.825 mg C-3-G/L of anthocyanins, and the sample with the addition of sucrose 286.553 mg C-3-G/L. The percentage of polymeric color was the same in all samples, around 30%, indicating that under the investigated conditions polymerization did not occur in the samples, and sugars had no effect on polymerization.

The results of the determination of antioxidant activity, by application of the DPPH (2,2-diphenyl picryl hydrazyl) and ABTS (2,2′-azino-bis(3-ethylbenzothiazoline-6-sulphonic acid) methods, of sour cherry juice with the addition of sugars are presented in Table 4. Sour cherry juice had the lowest antioxidant activity determined with both applied methods. The antioxidant activity of the control sample determined by the DPPH method was 1.115 µmol TE/100 mL. Samples with the addition of sugars had slightly higher antioxidant activity values, from 1.151 µmol TE/100 mL to 1.174 µmol TE/100 mL. The same tendency was retained with the ABTS method, only with higher antioxidant activity values. The antioxidant activity of sour cherry juice was 1.562 µmol TE/100 mL.

## 3. Discussion

The effect of sugar addition on the volatile compounds in different fruit products was investigated through several studies. A positive effect of trehalose on volatiles was observed for different samples depending on the properties of the volatile compounds, but also on the sample composition and conditions during processing and storage [13,14,15,16,17,18,19]. Also, there have been studies showing similar results on the protection of phenolics, flavonoids and anthocyanins by trehalose addition under different conditions during processing and storage [19,20,21,22,23]. In contrast to these results, in the study of Lončarić et al. [24] it was demonstrated that maltose also had a protective effect on anthocyanins in sour cherry puree, but those samples were prepared by freeze-drying.

In this study, juice was investigated as the food matrix, which in the highest percentage contains water. It is well known that water has a very specific and important role as a solvent and a reactant in many chemical reactions in foods. When water activity is high, reactions limited by diffusion are enhanced. Those reactions together with enzymatic activity, oxidation, and molecular mobility, which are all consequence of more available water, are causes of the degradation of sensitive compounds [25]. Anthocyanins are pigments soluble in water, thus water plays a major part in their degradation. The degradation of anthocyanins starts with the hydrolysis of the glycosidic bond in its structure and the formation of unstable anthocyanidins, followed by the opening of the pyrilium ring and the formation of chalcones and brown end products [26,27]. Also, in the presence of oxygen, water can increase the oxidation rate of anthocyanins. The investigated sugars (sucrose, maltose and trehalose) are chemical isomers, but as it is evident from the results on phenolics as well as volatiles, their behavior in a complex matrix can be different. Even if they are chemical isomers, they show different behavior in a much simpler system like water. Sugar molecules change water dynamics resulting in the effect of sugars on volatile and phenolic compounds. Trehalose binds to a larger number of water molecules than maltose and sucrose, leading to higher effect on water structure. Also, trehalose is able to form larger clusters than sucrose, but smaller than maltose. Lerbret et al. [28] suggested that trehalose–water mixtures are more homogeneous than sucrose or maltose water solutions based on the hydration number, the radius of gyration, the glycosidic dihedral angles and cluster formation. Steric hindrance developed by the disaccharide, which can protect or slow down the nucleophilic attacks of water that could cause the degradation of anthocyanins, were probably more pronounced in samples with trehalose addition because of the higher stability of trehalose, since trehalose structure is very stable regarding hydrolysis in comparison with sucrose [29]. Except for slowing down water dynamics, it is possible that some weak interactions occurred between trehalose and some of the compounds. The formation of stable intramolecular complexes between trehalose and unsaturated compounds that possess the *cis*-type olefinic double bond or with other structurally similar compounds [like benzene and p-cresol] is also a possible explanation of trehalose’s positive effect on phenolics and some volatiles [30,31,32]. These complexes can be formed with trehalose, but not with other disaccharides, due to the unique nature of trehalose, since the aromatic ring of compounds can approach the dehydrated, hydrophobic pocket of trehalose leading to the formation of the complex [32]. Volatiles are characterized by their diffusion. Sugars have different diffusion coefficients in water, but they also change the diffusion coefficient of water [33], thus, they are probably responsible for the change of the diffusion coefficient of volatile compounds and in this way for their retention in the fruit product matrix.

## 4. Materials and Methods

### 4.1. Sample Preparation

Sour cherry was obtained from local market and was kept at −20 °C prior to sample preparation. Juice was obtained by pressing the fruit and filtering it through cheesecloth. The obtained juice was mixed and homogenized with sugars (sucrose, trehalose or maltose) in the amount of 10% without any thermal treatment. The amount of sugars was selected on the basis of the most usual amount of sugars in industrial formulations for this type of product. Samples were left for stabilization in a glass jar for five days at room temperature and after that, a time analysis of phenolics and volatiles was conducted.

### 4.2. Gas Chromatography/Mass Spectrometry (GC/MS) Analysis

First SPME extraction of the compounds was conducted. A total of 8 g of sample was weighed in a glass vial, followed by 2.5 mL of saturated solution of CaCl_2_ and 40 µL of benzaldehyde-*d*_6_ solution (22.92 µg/100 mL) as internal standard. The extraction of volatiles was carried out using a solid-phase microextraction (SPME) fiber coated with divinylbenzene/carboxen/polydimethylsiloxane (DVB/CAR/PDMS) sorbent (1 cm long, 50/30 µm thickness, StableFlex™, Supelco, Bellefonte, PA, USA). The sample vials were conditioned in a temperature-controlled heating module at 40 °C for 45 min and agitated at 350 rpm. After extraction, the fiber was removed from the sample and the volatiles were thermally desorbed in the injector port of the GC.

The analysis of volatiles was conducted on a GC 7890A gas chromatograph (Agilent Technologies, Santa Clara, CA, USA) equipped with a MPS2 Multipurpose autosampler (Gerstel GmbH, Mülheim van der Ruhr, Germany) and 5975C mass spectrometer (Agilent Technologies). Volatile compounds were desorbed into a GC injector port at 250 °C in splitless mode for 2 min. The gas chromatograph was fitted with a ZB-WAX capillary column, 60 m × 0.32 mm i.d. with 1 μm film thickness. Helium was the carrier gas at a flow rate of 1.2 mL/min at 40 °C. The oven temperature was programmed as follows: initial temperature 40 °C held for 5 min, then 4 °C/min to 230 °C. The volatile compounds were identified with a mass selective detector (5975C, Agilent Technologies, Santa Clara, CA, USA). The detector operated in the *m/z* range between 30 and 250; the ion source and quadrupole temperature were maintained at 250 and 150 °C, respectively. The identification of compounds was performed by a comparison of their mass spectra with those of available commercial standards. All compounds were also confirmed by the matching of their mass spectra with the NIST 2.0 mass spectral database (National Institute of Standards and Technology, Gaithersburg, MD, USA). Internal standard (benzaldehyde-d6) was used for quantification of volatiles and amount of volatiles were expressed as µg/100 mL of juice. Five repetitions were conducted for each sample.

### 4.3. Determination of Total Phenolics

The concentration of total polyphenols was estimated by the Folin–Ciocalteau method [34]. For each sample, the measurements were performed in triplicate and the values were interpolated on a gallic acid calibration curve and expressed as g of gallic acid equivalents per L of sample (g GAE/L).

### 4.4. Determination of Flavonoids and Flavonols

Flavonoids and flavonols were determined according to Kumaran and Karunakaran [35]. For each sample, the measurements were performed in triplicate and the values (for flavonoids and flavonols) were interpolated on a rutin calibration curve and expressed as mg of rutin equivalents per L of sample (mg RE/L).

### 4.5. Total Monomeric Anthocyanin Content and Polymeric Color Determination

Total monomeric anthocyanin content and polymeric color were determined by the method described by Giusti and Wrolstad [36]. Monomeric anthocyanins were determined by pH-differential method and anthocyanins were expressed as cyanidin-3-glucoside.

### 4.6. Antioxidant Activity

The antioxidant activity of the samples was determined by two different methods. For the ABTS method, sample (0.2 mL) reacted with 3 mL of the ABTS radical solution and the absorbance was taken at 734 nm after 95 min using a spectrophotometer. For blank the extract was replaced with water. The DPPH radical scavenging assay was performed by mixing the 0.2 mL of extract with 3 mL of DPPH solution. For blank the extract was replaced with water. The reaction mixture was vortexed thoroughly and left in the dark at room temperature for 15 min. After 15 min the absorbance was measured at 517 nm. For each assay, the sample measurements were performed in triplicate and values were interpolated on a trolox calibration curve and expressed as µmol of trolox equivalents per 100 mL of sample (µmol TE/100 mL).

### 4.7. Statistical Analysis

The results are expressed as the mean values ± standard deviation. The data of the volatile compounds amount were analyzed by analysis of variance (ANOVA) and Fisher’s least significant difference (LSD) with the significance defined at *p* < 0.05. All statistical analyses were carried out using the software program STATISTICA 7 (StatSoft Inc., Palo Alto, CA, USA).

## 5. Conclusions

The results of the evaluation of the effect of disaccharide addition to sour cherry juice showed that the type of disaccharide had an important impact on phenolics and volatiles. Overall, trehalose had a higher positive impact on volatiles, with a desirable flavor note, as well as a positive impact on phenolics and anthocyanins. These results can be used for the formulation of products that are less sweet and have possible health benefits.

## Figures and Tables

**Table 1:** Volatile compounds (µg/100 mL) of sour cherry juice with the addition of sugars.

Volatiles	Juice	Juice + Maltose	Juice + Sucrose	Juice + Trehalose
1-propanol	4.365±0.205 ^a,c^	3.923±0.129 ^b^	4.472±0.054 ^c^	4.113±0.087 ^a,b^
2-methyl-1-propanol	14.335±0.583 ^a^	9.079±0.335 ^b^	12.0258±0.452 ^c^	10.744±0.265 ^d^
1-butanol	29.727±0.949 ^a,b^	29.354±0.328 ^a^	31.331±0.656 ^b^	29.373±0.493 ^a^
3-methyl-1-butanol	3.255±0.241 ^a^	2.507±0.122 ^b^	2.825±0.044 ^c^	2.707±0.071 ^c^
3-methyl-3-buten-1-ol	0.278±0.018 ^a^	0.144±0.020 ^b^	0.219±0.013 ^c^	0.186±0.026 ^b^
1-pentanol	1.163±0.030 ^a^	1.931±0.152 ^b^	1.863±0.049 ^b^	1.615±0.069 ^c^
1-penten-3-ol	0.064±0.015 ^a^	0.059±0.008 ^a^	0.065±0.005 ^a^	0.078±0.011 ^a^
1-hexanol	5.730±0.129 ^a^	5.769±0.190 ^a^	6.196±0.086 ^b^	6.278±0.120 ^b^
2-heptanol	5.087±0.114 ^a^	5.265±0.160 ^a,b^	5.371±0.120 ^b^	5.423±0.131 ^b^
1-octanol	0.785±0.031 ^a^	0.469±0.094 ^b^	1.032±0.080 ^c^	0.891±0.065 ^d^
benzyl alcohol	3.477±0.170 ^a^	0.0048±0.001 ^b^	4.207±0.130 ^c^	4.050±0.222 ^c^
2-phenyl ethyl alcohol	3.556±0.320 ^a^	0.261±0.028 ^b^	0.422±0.096 ^c^	0.444±0.039 ^c^
1-butanol-3-metyl-acetate	1.407±0.052 ^a^	1.555±0.315 ^a,b^	1.641±0.095 ^a,b,c^	1.844±0.102 ^c^
hexyl acetate	0.647±0.041 ^a^	0.630±0.054 ^a^	0.788±0.057 ^b^	0.933±0.053 ^c^
2-methyl butyl butanoate	1.061±0.106 ^a^	1.340±0.116 ^b^	0.932±0.069 ^a^	1.292±0.121 ^b^
3-methyl butyl butanoate	0.216±0.016 ^a,b^	0.187±0.013 ^a^	0.231±0.017 ^b^	0.290±0.018 ^c^
2-metyl butanoate	1.616±0.035 ^a^	1.864±0.043 ^b^	1.902±0.010 ^b^	1.923±0.075 ^b^
2-hexenal	0.467±0.018 ^a^	0.469±0.032 ^a,b^	0.501±0.015 ^b^	0.523±0.024 ^b^
β-cyclocitral	0.897±0.036 ^a^	0.842±0.044 ^a^	1.011±0.050 ^b^	1.055±0.031 ^b^
benzaldehyde	85.901±0.260 ^a^	77.214±3.053 ^b^	81.023±2.664 ^b^	84.716±0.264 ^c^
α-ionone	3.979±0.125 ^a^	3.425±0.226 ^b^	3.518±0.197 ^b^	3.797±0.135 ^a,b^
β-ionone	5.111±0.226 ^a^	4.461±0.112 ^b^	4.480±0.130 ^b^	4.946±0.154 ^a^
diacetyl	0.949±0.045 ^a^	0.881±0.048 ^a^	0.906±0.022 ^a^	0.904±0.013 ^a^
2-decanon	0.869±0.073 ^a^	0.909±0.021 ^a^	0.855±0.070 ^a^	0.931±0.027 ^a^
acetoin	0.736±0.044 ^a^	0.749±0.044 ^a^	0.813±0.042 ^a^	0.769±0.038 ^a^
o-cymene	8.959±0.712 ^a^	12.271±0.488 ^b^	14.142±0.146 ^c^	12.149±0.215 ^b^
geranic oxide	2.991±0.525 ^a^	8.596±0.565 ^b^	4.969±0.377 ^c^	7.401±0.619 ^b^
eugenol	0.473±0.010 ^a^	0.600±0.031 ^b^	0.582±0.022 ^b^	0.623±0.041 ^b^

Values in the same row followed by different letters are significantly different at *p* < 0.05, (ANOVA, Fisher’s LSD).

**Table 2:** Phenolics, flavonoids and flavonols of sour cherry juice with the addition of sugars.

Samples	Phenolic Content (g GAE/L)	Flavonoid Content (mg RE/L)	Flavanone Content (mg RE/L)
juice	1.550 ± 0.014 ^a^	354.84 ± 0.05 ^a^	121.11 ± 0.04 ^a^
juice + sucrose	1.600 ± 0.018 ^b^	369.62 ± 0.02 ^b^	79.84 ± 0.06 ^b^
juice + trehalose	1.633 ± 0.011 ^b^	330.19 ± 0.03 ^c^	28.33 ± 0.08 ^b^
juice + maltose	1.443 ± 0.016 ^c^	378.49 ± 0.04 ^d^	161.98 ± 0.08 ^c^

GAE—gallic acid equivalents; RE—rutin equivalents; Values in the same row followed by different letters are significantly different at *p* < 0.05, (ANOVA, Fisher’s LSD).

**Table 3:** Anthocyanin content and polymeric color of sour cherry juice with the addition of sugars.

Samples	Anthocyanin Content (mg C-3-G/L)	Percentage of Polymeric Color (%)
juice	214.038 ± 1.199 ^a^	29.375 ± 0.148 ^a^
juice + sucrose	286.553 ± 1.416 ^b^	29.995 ± 0.155 ^a^
juice + trehalose	297.825 ± 1.250 ^c^	30.405 ± 0.186 ^a^
juice + maltose	206.649 ± 1.896 ^d^	29.456 ± 0.147 ^a^

C-3-G—cyanidin-3-glucoside equivalents; Values in the same row followed by different letters are significantly different at *p*< 0.05, (ANOVA, Fisher’s LSD).

**Table 4:** Antioxidant activity of sour cherry juice with the addition of sugars.

Samples	DPPH	ABTS
juice	1.115 ± 0.008 ^a^	1.562 ± 0.006 ^a^
juice + sucrose	1.151 ± 0.010 ^b^	1.662 ± 0.008 ^b^
juice + trehalose	1.174 ± 0.006 ^c^	1.680 ± 0.005 ^c^
juice + maltose	1.165 ± 0.006 ^b,c^	1.672 ± 0.005 ^b,c^

Values in the same row followed by different letters are significantly different at *p* < 0.05, (ANOVA, Fisher’s LSD).
